# Molecular detection and genotypic characterization of *Toxoplasma gondii* infection in bats in four provinces of China

**DOI:** 10.1186/s13071-014-0558-7

**Published:** 2014-12-03

**Authors:** Si-Yuan Qin, Wei Cong, Ye Liu, Nan Li, Ze-Dong Wang, Fu-Kai Zhang, Si-Yang Huang, Xing-Quan Zhu, Quan Liu

**Affiliations:** State Key Laboratory of Veterinary Etiological Biology, Key Laboratory of Veterinary Parasitology of Gansu Province, Lanzhou Veterinary Research Institute, Chinese Academy of Agricultural Sciences, Lanzhou, Gansu Province 730046 PR China; Military Veterinary Institute, Academy of Military Medical Sciences, Key Laboratory of Jilin Province for Zoonosis Prevention and Control, Changchun, Jilin Province 130122 PR China; College of Animal Science and Technology, Jilin Agricultural University, Changchun, Jilin Province 130118 PR China; Jiangsu Co-innovation Center for the Prevention and Control of Important Animal Infectious Diseases and Zoonoses, Yangzhou University College of Veterinary Medicine, Yangzhou, Jiangsu Province 225009 PR China

**Keywords:** *Toxoplasma gondii*, Bats, Prevalence, Genetic characterization, China

## Abstract

**Background:**

*Toxoplasma gondii* is an intracellular protozoan parasite that infects a wide variety of warm-blooded hosts, including humans. Limited information about *T. gondii* infection in bats is available in China. The objective of the present study was to determine prevalence and genetic characterization of *T. gondii* infection in bats in Jilin, Liaoning, Jiangxi and Guangdong provinces, China.

**Methods:**

During May 2005 to August 2013, bats were sampled from Jilin, Liaoning, Jiangxi, and Guangdong provinces, China, and liver tissues were collected for the detection of *T. gondii* by a nested PCR targeting the B1 gene. The positive samples were genotyped at 11 genetic markers (SAG1, 5′-and 3′-SAG2, alternative SAG2, SAG3, BTUB, GRA6, L358, PK1, c22-8, c29-2, and Apico) using multilocus polymerase chain reaction-restriction fragment length polymorphism (PCR-RFLP).

**Results:**

A total of 626 bats representing 10 species were examined for *T. gondii* infection, 38 (6.1%) were tested positive with by PCR, 8 positive DNA samples were completely genotyped, of which 3 samples (2 from *Cynopterus sphinx*, and 1 from *Murina leucogaster*) represented ToxoDB#10, and 5 samples (2 from *Murina leucogaster*, 2 from *Myotis chinensis*, and 1 from *Rhinolophus ferrumequinum*) belonged to ToxoDB#9 (http://toxodb.org/toxo/).

**Conclusions:**

The present study revealed an overall *T. gondii* prevalence of 6.1% in bats from Jilin, Liaoning, Jiangxi and Guangdong provinces in China, and reported two *T. gondii* genotypes (ToxoDB#9 and #10) having a wide geographical distribution in China. These results provide new genetic information about *T. gondii* infection in bats, and have implications for better understanding of the genetic diversity of *T. gondii* in China and elsewhere.

## Background

*Toxoplasma gondii* is an obligate intracellular protozoan parasite with a worldwide distribution, which can infect a wide variety of warm blooded animals and humans. Approximately one third of the world population and 7.9% of population in China are seropositive for *T. gondii* antibodies [1-3]. *T. gondii* infection can cause serious diseases in the developing fetus and immunocompromised individuals [4]. It is transmitted to humans and animals via ingesting *T. gondii* tissue cysts from undercooked meat, by consuming water or food contaminated with *T. gondii* oocysts, or through transplacental transmission [5,6].

Bats are considered as important natural reservoir of many zoonotic viruses, such as rabies virus, Hantavirus, Marburg virus, Nipah virus, Ebola virus and severe acute respiratory syndrome coronaviruses [7,8]. Besides viruses, bacteria, fungi, and protozoa have been detected in bats, and can potentially be transmitted to humans [9-11]. Bats are important to public health, as they live in different habitats and have high mobility and the possible interactions with humans.

Although bats play significant roles in the transmission of some important zoonotic diseases, there were only limited reports on *T. gondii* infection in bats. The prevalence of *T. gondii* infection was 10.4% in British bats [12], and 29.3% in bats in Myanmar [13]. Additionally, there were two cases of toxoplasmosis described in captive bats in Australia [14], and several *T. gondii* strains have been isolated in Kazakhstan and in Brazil [15,16]. In China, *T. gondii* seroprevalence in five bat species in four provinces was detected using modified agglutination test (MAT) [17]. However, there was only one previous report on genotypes of *T. gondii* in bats in Yunnan and Guangxi, southern China [18]. In the present study, we determined the prevalence and characterized *T. gondii* isolates in bats in other four provinces of China.

## Methods

### Ethics statement

This study was approved by the Ethics Committee of Military Veterinary Institute, Academy of Military Medical Sciences. Bats were handled in accordance with good animal practices required by the Animal Ethics Procedures and Guidelines of the People’s Republic of China.

### Study sites and bat collection

The study was conducted in four provinces of China, including Jilin (41°–46° N, 122°–131° E), Liaoning (38°–43° N, 118°–125° E), Jiangxi (24°–30° N, 113°–118° E), and Guangdong (20°–25° N, 109°–117° E) from May 2005 to August 2013. Jilin and Liaoning provinces are located in northeastern of China, where the average temperature is lower than other two provinces. Jiangxi province, located in southeast China, has an average annual temperature of 11.6–19.6°C. Guangdong province lies on southern edge of mainland, with an average annual temperature of 22.8°C. Bats were captured at roosts with hand nets, and identified to species in the field.

### DNA extraction and PCR detection

Bats were euthanized, and liver tissues were collected for genomic DNA extraction using the TIANamp Genomic DNA kit (TianGen, Beijing, China). *T. gondii* infection was examined by a semi-nested PCR targeting the B1 gene as described elsewhere [19-21]. The positive samples were used further for genetic characterization.

### Genetic characterization of *T. gondii*

Multilocus PCR-RFLP was conducted to genetically characterize *T. gondii* in bats, using 11 genetic markers (i.e., SAG1, 5′-and 3′-SAG2, alternative SAG2, SAG3, BTUB, GRA6, c22-8, c29-2, L358, PK1, and Apico) as described previously [12,18]. Briefly, the target sequences were first amplified by multiplex PCR using external primers for all 11 markers. Nine reference strains, namely GT1, PTG, CTG, MAS, TgCgCa1, TgCatBr5, TgCatBr64, TgRsCr1, and TgWtdSc40, were used as the positive controls (Table 1). The PCR amplification was performed using a thermal cycler (PTC 200, Bio-RAD). The *T. gondii* B1-positive DNA sample was incubated at 95°C for 5 min to activate the DNA polymerase, then 30 cycles of PCR at 95°C for 30 s, 55°C for 60 s and 72°C for 90 s, and then at 72°C for 7 min. Then 1 μl of the products were used as template DNA for nested PCR with internal primers for each marker, respectively. The nested PCR products were digested with restriction enzymes for 2 h, and the restriction fragments were resolved in 2.5% agarose gel to distinguish single nucleotide polymorphisms (SNPs) using a gel document system (UVP GelDoc-It™ Imaging System, Cambridge, UK).

**Table 1:** **Genetic characterization of** ***Toxoplasma gondii*** **isolates from bats in Jilin, Jiangxi and Guangdong provinces, China**

**Isolate ID**	**Host**	**Tissue**	**Location**	**SAG1**	**5′ + 3′ SAG2**	**Alternative SAG2**	**SAG3**	**BTUB**	**GRA6**	**c22-8**	**c29-2**	**L358**	**PK1**	**Apico**	**Genotype**
GT1	Goat		United States	I	I	I	I	I	I	I	I	I	I	I	Reference, Type I, ToxoDB #10
PTG	Sheep		United States	II/III	II	II	II	II	II	II	II	II	II	II	Reference, Type II, ToxoDB #1
CTG	Cat		United States	II/III	III	III	III	III	III	III	III	III	III	III	Reference, Type III, ToxoDB #2
MAS	Human		France	u-1^*^	I	II	III	III	III	u-1^*^	I	I	III	I	Reference, ToxoDB #17
TgCgCa1	Cougar		Canada	I	II	II	III	II	II	II	u-1^*^	I	u-2^*^	I	Reference, ToxoDB #66
TgCatBr5	Cat		Brazil	I	III	III	III	III	III	I	I	I	u-1^*^	I	Reference, ToxoDB #19
TgWtdSc40	WTD		USA	u-1	II	II	II	II	II	II	II	I	II	I	Type 12, ToxoDB #5
TgCatBr64	Cat		Brazil	I	I	u-1	III	III	III	u-1	I	III	III	I	Reference, ToxoDB #111
TgRsCr1	Toucan		Costa Rica	u-1	I	II	III	I	III	u-2	I	I	III	I	Reference, ToxoDB #52
TgBatJL1- TgBatJL2	MC	Liver	Jilin, China	u-1	II	II	III	III	II	II	III	II	II	I	ToxoDB #9
TgBatJL3	ML	Liver	Jilin, China	I	I	I	I	I	I	I	I	I	I	I	ToxoDB #10
TgBatJL4	ML	Liver	Jilin, China	I	I	I	I	nd	I	I	I	I	nd	nd	=nd
TgBatJX5- TgBatJX7	RF	Liver	Jiangxi, China	u-1	II	II	III	III	II	II	III	II	II	I	ToxoDB #9
TgBatGD8	CS	Liver	Guangdong, China	I	I	I	I	I	I	I	nd	I	I	nd	nd
TgBatGD9, TgBatGD10	CS	Liver	Guangdong, China	I	I	I	I	I	I	I	I	I	I	I	ToxoDB #10
TgBatGD11	CS	Liver	Guangdong, China	nd	I	nd	I	I	I	I	I	I	nd	nd	nd

*u-1 and u-2 represent unique RFLP genotypes, respectively.

nd: not determined.

WTD: White-tailed Deer; CS: *Cynopterus sphinx*; ML: *Murina leucogaster*; MC: *Myotis chinensis*; RF: *Rhinolophus ferrumequinum*.

## Results and discussion

A total of 626 bats, belonging to ten species of seven genera, were collected in the present study (Table 2). The dominant bat species was *Murina leucogaster* in Jilin, *Myotis ricketti* in Liaoning, *M. leucogaster* and *Myotis chinensis* in Jiangxi, and *Hipposideros larvatus* and *Cynopterus sphinx* in Guangdong.

**Table 2:** **Prevalence of** ***Toxoplasma gondii*** **infection in bats in four provinces, China**

**Province**	**Bat species**	No. of examined (%)*	**No. of positive**	**Prevalence (%)**
Jilin	*Murina leucogaster*	140 (22.4)	6	4.3
	*Myotis chinensis*	27 (4.3)	3	11.1
	*Plecotus auritus*	2 (0.3)	0	0.0
	*Rhinolophus ferrumequinum*	8 (1.3)	1	12.5
	Subtotal	177 (28.3)	10	5.6
Liaoning	*Myotis chinensis*	9 (1.4)	0	0.0
	*Myotis ricketti*	56 (8.9)	2	3.6
	*Rhinolophus ferrumequinum*	17 (2.7)	1	5.9
	Subtotal	82 (13.1)	3	3.7
Jiangxi	*Murina leucogaster*	82 (13.1)	2	2.4
	*Myotis chinensis*	103 (16.5)	7	6.8
	*Rhinolophus ferrumequinum*	18 (2.9)	4	22.2
	Subtotal	203 (32.4)	13	6.4
Guangdong	*Cynopterus sphinx*	54 (8.6)	1	1.9
	*Hipposideros armiger*	4 (0.6)	0	0.0
	*Hipposideros larvatus*	67 (10.7)	8	11.9
	*Hipposideros pomona*	9 (1.4)	0	0.0
	*Rousettus leschenaulti*	30 (4.8)	3	10.0
	Subtotal	164 (26.2)	12	7.3
Total		626	38	6.1

*The percent accounts for the total bats.

Of 626 examined bat samples, 38 samples were tested positive for the *T. gondii* B1 gene by PCR amplification, including 8 of 222 (3.6%) *M. leucogaster*, 10 of 139 (7.2%) *M. chinensis*, 2 of 56 (3.6%) *M. ricketti*, 6 of 43 (14.0%) *Rhinolophus ferrumequinum*, 1 of 54 (1.9%) *Cynopterus sphinx*, 3 of 30 (10.0%) *Rousettus leschenaultia*, 8 of 67 (11.9%) *H. larvatus* (Table 2). Only three species, including *Plecotus auritus* in Jilin, *M. chinensis* in Liaoning, and *Hipposideros armiger* in Guangdong, were detected negative, probably due to the small number of samples, or the low prevalence.

The results of the present study demonstrated that *T. gondii* infection in bats was widely distributed in Jilin, Liaoning, Jiangxi and Guangdong provinces, with prevalence ranging from 3.7% to 7.3%. Other surveys have shown an overall prevalence of 6.7% in bats in Yunnan, and 20.3% in Guangxi by a nested PCR [18], and a seroprevalence of 18.4% in five bat species in Anhui, Hubei, Guangdong, and Guangxi by MAT [17]. In other countries, the prevalence of *T. gondii* infection was found in 10.4% British bats using a SAG1-PCR [12], and in 29.3% bats in Myanmar by a nested B1-PCR [13]. The difference may be related to bats species, study regions, and the detection methods.

The present study revealed that *T. gondii* prevalence was higher in bats in southern China (Guangdong, and Jiangxi) than in northern China (Jilin, and Liaoning). The possible reason is that warm and humid environment in southern China is more suitable for survival of *T. gondii* oocysts [1].

The 38 *T. gondii*-positive bat samples were directly genotyped, and only 8 positive DNA samples were completely genotyped, possibly due to low DNA concentration. Of which, 3 samples (2 from *C. sphinx*, and 1 from *M. leucogaster*) represented ToxoDB Genotype #10, and 5 samples (2 from *M. leucogaster*, 2 from *M. chinensis*, and 1 from *R. ferrumequinum*) belonged to ToxoDB Genotype #9 (Table 1).

Two genotypes, namely ToxoDB#10 and ToxoDB#9, were found in bats in this study, which was consistent with a previous study [18], showing a limited diversity of *T. gondii* genotypes in bats in China. Unfortunately, *T. gondii* genotype in bats was not successfully identified in Liaoning, probably due to the small size of sampled bats and low intensity of infection.

Recent studies have demonstrated that bats may share the same *T. gondii* genotypes as in wild and domestic animals, and humans. Although several genotypes of *T. gondii* have been described in China, there are two main genotypes, including types I (ToxoDB#10) and an atypical genotype ToxoDB#9. In particular, genotype #9 has been reported in *Microtus fortis* in Jilin province [22], pigs in Jiangxi and Yunnan provinces [20,23], cats in Beijing, Yunnan and Guangdong provinces [24-26], bats in Yunnan and Guangxi provinces [18], and humans [27,28], suggesting that the genotype ToxoDB#9 is the most common lineage in mainland China. It is not only present in China, but also in Vietnam and Sri Lanka, which indicated a widespread distribution in Eastern Asia [29]. ToxoDB#10 is also common in China, found in Plateau pikas and Qinghai voles in Qinghai [19], *M. fortis* in Jilin [22], tree sparrows in Fujian [30], pigs in Henan, Hubei, Hunan, and Jiangsu [27,31], sheep in Qinghai [27], and human in Shanghai [27]. These results have shown a wide distribution of the two genotypes identified from bats in China.

## Conclusions

The present study revealed an overall *T. gondii* prevalence of 6.1% in bats from Jilin, Liaoning, Jiangxi and Guangdong provinces, China, and reported two *T. gondii* genotypes (ToxoDB#9 and #10). The wide geographical distribution of the two genotypes implied an important role of bats in transmission of *T. gondii*. These results provide new genetic information about *T. gondii* infection in bats, and have implications for better understanding of the genetic diversity of *T. gondii* in China.
